# Parasitic Helminths in Wild Boars (*Sus scrofa*) in Mazandaran Province, Northern Iran

**Published:** 2018

**Authors:** Samira DODANGEH, Davoud AZAMI, Ahmad DARYANI, Shirzad GHOLAMI, Mehdi SHARIF, Iraj MOBEDI, Shahabeddin SARVI, Eissa SOLEYMANI, Mohammad Taghi RAHIMI, Majid PIRESTANI, Shaban GOHARDEHI, Reza BASTANI

**Affiliations:** 1. Toxoplasmosis Research Center, Mazandaran University of Medical Sciences, Sari, Iran; 2. Student Research Committee, Mazandaran University of Medical Sciences, Sari, Iran; 3. Dept. of Medical Parasitology and Mycology, School of Public Health, Tehran University of Medical Sciences, Tehran, Iran; 4. Dept. of Basic Medical Sciences, School of Medicine, Shahroud University of Medical Sciences, Shahroud, Iran; 5. Dept. of Parasitology, School of Medical Sciences, Tarbiat Modares University, Tehran, Iran

**Keywords:** Wild boar, Parasitic helminths, Prevalence, Iran

## Abstract

**Background:**

Wild boars (*Sus scrofa*) are distributed worldwide and found in many parts of Iran. Although *S. scrofa* is reservoirs for many parasites, there is little data on helminthic prevalence in them. We aimed to survey the status of helminthic infections in *S. scrofa* in the Mazandaran Province of northern Iran.

**Methods:**

Twenty-one wild boars were captured and examined for helminth infection during Dec 2012–Mar 2014. Adult worms such as *Macracanthorhynchus hirudinaceus* were identified by helminth size and shape, and the arrangement of the proboscis hooks. The sedimentation and flotation techniques were used to detect parasite eggs and larvae in faecal samples. Muscle samples were also surveyed for *Trichinella* larvae by artificial digestion method.

**Results:**

Of the 21 samples, 13 (61.9%) were infected with one or more helminth species. Seven helminth types were identified in the alimentary track, comprising 5 nematodes, 1 trematode, and 1 acanthocephalan, with prevalence rates of *Macracanthorhynchus hirudinaceus* (57.14%)*, Globocephalus* spp. (33.33%)*, Trichuris suis* (19.04), *Gongylonema pulchrum* (14.28%), *Fasciola hepatica* (14.28%), *Dioctophyma renale* (4.76%), and *Ascaris suum* (4.76%).

**Conclusion:**

Wild boars might be involved in transmitting zoonotic parasites to humans. The abundance of these animals near human habitation creates favorable conditions for infection. So the risk of parasitic helminth diseases increases in other animals and humans.

## Introduction

Wild boars (*Sus scrofa*) are distributed worldwide (1) and are omnivorous, with diets comprising insect larvae, amphibians, reptiles, mushrooms, birds, bird eggs, small rodents, fruits, etc. (2). Owing to their natural feeding habits, *S. scrofa* is host and reservoir of many parasites; they play an important role in the transmission of infections to domestic animals and humans (2).

*Sus scrofa* may pass feces including infectious agents in the farming fields and cause to contaminate water resources and the subsequent these agents influence to crops and plants. Therefore, people who eat these plants are infected (3).

Wild boars are found in many parts of Iran, especially in the mountainous areas. They usually cause destruction of crops and are often killed by farmers or hunted for meat by Christian Armenians (3). Although *S. scrofa* is widely distributed in northern Iran, little information is available about the parasitic infections in wild boars in northern Iran.

We determined the status of helminthic infections in wild boars in the Mazandaran Province of northern Iran.

## Materials and Methods

### Ethical approval

Ethical approval of present study was received from the Ethics Committee in Mazandaran University of Medical Sciences, Mazandaran, Iran.

### Study area

**Fig. 1:**
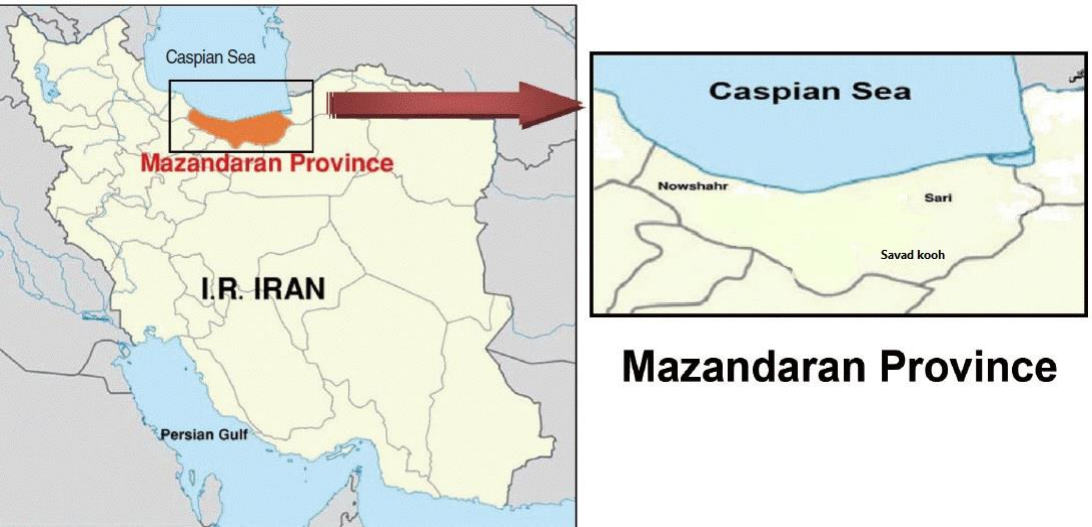
Map of Mazandaran Province, northern Iran and 3 different cities (Sari, Savadkuh, and Behshahr), Iran

This study was conducted in the suburbs of 3 different cities (Sari, Savadkuh, and Behshahr) in the Mazandaran Province, Iran. The Mazandaran province (36° 33′ 56″ N 53° 03′ 32″ E) is located in northern Iran on the southern coast of the Caspian Sea (Fig. 1). It covers an area of approximately 23842 km^2^ and has a population of 2,922,432 individuals. This province has a moderate and subtropical climate and is geographically divided into the three regions: plains, forests, and mountains.

### Sample collection

Twenty-one wild boars were captured from Dec 2012 to Mar 2014. During sampling, the shooting site, sampling time, sex, and age of each boar were recorded. Based on tooth shape and development and physical appearance, the samples were categorized into three age groups (4). The abdominal viscera of the animals were removed immediately after capture, placed in plastic bags, labeled, and sent to the parasitology laboratory, Mazandaran University, Iran. Necropsy was performed in the laboratory, and in the first step, internal organs such as oesophagus, stomach, small and large intestines, liver, and muscles, including the diaphragm were examined macroscopically for the presence of helminthic parasites. All the parasite worms were identified (5). For example in present study, *M. hirudinaceus* was identified according to helminth size and shape, and the arrangement of the probosci’s hooks.

To recover non-visible helminths, the stomach and intestine were separately washed in buckets with tap water and squeezed through 500-μm and 150-μm sieves. The sedimentation and flotation techniques were used to detect parasite eggs and larvae in faecal samples collected from the intestinal tract (6). The ova were identified based on morphological features (shape, structure, and presence of larva) (7). In addition, muscle samples were surveyed for *Trichinella* larvae by two direct and artificial digestion methods.

### Statistical analysis

SPSS version 16.0 (Chicago, IL, USA) was used for all statistical analyses. Chi-squared (χ^2^) test was performed to investigate the significance of the differences in prevalence of helminth infections between age and sex in the different groups. The level of significance was *P*<0.05 for all calculations.

## Results

**Fig. 2:**
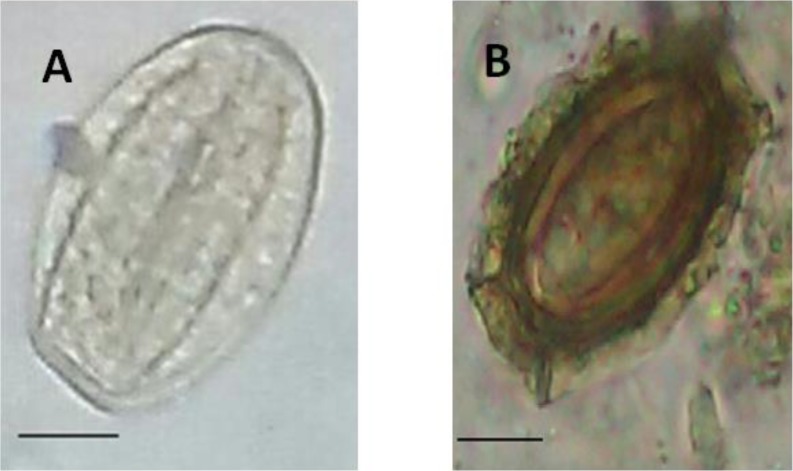
Endoparasites of isolated from *Sus scrofa* A: Egg of *Macracanthorhynchus hirudinaceu*s; B: Egg of *Trichuris suis*. Bars=15 μm. Eggs are shown by ×40 magnification (Original)

Of the 21 wild boars (11 females and 10 males) examined for helminthic infections, 13 (61.9%) were infected with one or more helminth species. Seven helminth types were identified, comprising 5 nematodes, 1 trematode, and 1 acanthocephalan. The prevalence of helminth species in the suburbs of 3 different cities in the Mazandaran Province are shown in Table 1. The highest prevalence was observed for *M. hirudinaceu*s (57.14%) (Fig. 2), while the lowest was recorded for *Dioctophyma renale* (4.76%) and *Ascaris suum* (4.76%). Helminth infections were found in 10 of 11 wild boars (90.9%) in Savadkuh, 3 of 5 (60.0%) in Behshahr, and 0 of 5 (0%) in Sari. Of the 13 (61.9%) wild boars infected with helminth species, Poly-parasitism was more common (84.62%) than mono-parasitism (15.38%). Poly-parasitism with *M. hirudinaceus* was the most frequent (Table 2). The age of the wild boars could not influence the prevalence of infection. In addition, there was no significant difference in the prevalence rates of infection in males and females (Table 3).

**Table 1:** Prevalence of helminth infections in *Sus scrofa* in Mazandaran, northern Iran

***Parasites***	***Infected wild boars number***			***Prevalence (%)***
	**Behshahr City**	**Sari City**	**Savadkuh City**	
*M. hirudinaceus*	9	0	3	57.14
*Globocephalus* spp.	7	0	0	33.33
*T. suis*	3	0	1	19.04
*G. pulchrum*	3	0	0	14.28
*F. hepatica*	2	0	1	14.28
*Dioctophyma renale*	1	0	0	4.76
*A. suum*	1	0	0	4.76

**Table 2:** Prevalence of multiple infections (helminths) in 21 wild boars in Mazandaran, northern Iran

***Species of parasite per wild boar***	***Number of infected wild boars***
*M. hirudinaceus*	2
*M. hirudinaceus + Globocephalus* spp.	2
*M. hirudinaceus + T. suis*	1
*M. hirundinaceus + F. hepatica*	1
*Globocephalus* spp. *+ A. suum*	1
*M. hirudinaceus + Globocephalus* spp. *+ G. pulchrum*	2
*M. hirudinaceus + Globocephalus* spp. *+ T. suis*	1
*M. hirudinaceus + T. suis + F. hepatica*	1
*M. hirudinaceus + F. hepatica + G. pulchrum*	1
*M. hirudinaceus + Globocephalus spp. + T. suis + Dioctophyma renale*	1
Total number of infected wild boars	13

**Table 3:** Prevalence of helminth infections of wild boars in relation with age and sex

***Variable***	***Number examined***	***Number infected***	***Percentage (%)***	***P-value***
**Age (yr)**				> 0.05
≤ 1	9	3	33.3	
1< and <3	6	4	66.6	
3–10	6	6	100	
**Sex**				> 0.05
Male	10	8	80	
Female	11	5	45.4	
Total	21	13	61.9	

## Discussion

There is little data available on helminth prevalence in *S. scrofa* in Iran, and there have been no comprehensive studies on worm prevalence in the Mazandaran Province. The results of this study showed a helminth prevalence of 61.9% in 21 *S. scrofa* from the Mazandaran Province of Iran. This prevalence was higher than that reported from Talesh City, in the Guilan Province of Iran (34%) (8), but was almost similar to results (1), who reported a prevalence of 74% in the north, northeast, and southwest of Iran, and another (9), who reported a prevalence of 58.3% in Luristan in western Iran. Similarly, in Tamil Nadu, south India opined which majority of wild boars surveyed (62%) had at least one helminth species in the internal organs (10). The reasons for such similarity in Tamil Nadu and Mazandaran might be assigned to the feeding regime of wild boars and climatic conditions of these two areas.

In the present study, we found seven types of helminths in wild boars, while 16 species of worms were reported in 57 wild boars (1). Of the 7 important nematode species included in the veterinary report (11), two were found in our study: *A. suum* and *T. suis*.

Our results also showed that *M. hirudinaceus* was the most prevalent helminth, followed by *Globocephalus* spp. While this is consistent with the other results (9), it is in contrast to the results (1), who reported that *Globocephalus* spp. was the most prevalent helminth in wild boars. *M. hirudinaceus* is distributed worldwide and has been reported in different hosts such as dogs, pigs, and even humans (12). While this parasite has been frequently reported in kinds of dogs and wild boars in Iran (13), human infection has not yet been reported. Definitive hosts have been known to be infected after ingestion of different beetle species (14). In this study, 12 of the 21 (57.14%) *S. scrofa* were infected with this parasite, indicating that these animals could play an important role in the infection of humans with *M. hirudinaceus* in Iran, especially in the suburban and rural areas.

In this study, the prevalence rate of 33.33% was recorded for *Globocephalus* spp. The moderately high prevalence of *Globocephalus* spp. might be due to the suitable conditions for resistance against the infectious larvae in this region. Wild boars infected with *Globocephalus* spp. suffer from anaemia and pathologic changes in the mucosa (15).

*T. suis* (Fig. 2), found in this study, is an intestinal nematode found in wild boars (16), and is similar to *Trichuris trichura* in humans, with respect to life cycle, morphology, and symptoms (17). Additionally, the successful transmission of this nematode from pigs to humans was reported (18).

*G. pulchrum* is another nematode identified in the present study with a prevalence of 14.28%. A higher prevalence (35%) of this worm was reported (1). Humans could be accidentally infected with *G. pulchrum* (19).

*F. hepatica*, a liver fluke identified in the current study with a prevalence of 14.28%, was reported to have a prevalence of 4% (1). In the northwest region of Spain, a prevalence of 11.2% for *F. hepatica* was found in 358 wild boars from Galicia (20). This was the first study to suggest that wild boars could be primary hosts for *F. hepatica*. As people consume forest herbs and plants in the northern regions of Iran, wild boars might be considered reservoir host of fascioliasis for human infection.

Among the helminths identified in this study, egg *Dioctophyma renale* was also observed in the faeces; while eggs of mentioned species are shed in the urine, there is the possibility that the eggs passed through the alimentary system of wild boar after the consumption of infected host.

Furthermore, the prevalence of 4.76% recorded for *A. suum* in this survey was almost the same as that reported in three geographical areas (north, northeast, and southwest) of Iran (5%) (1), but was lower than that reported in Eastern Ghana (12.7%) (21) and Botswana (54.6%) (22). Although the prevalence of *A. suum* recorded in the present study was not very high, it is nevertheless important because this parasite has been known to cause severe visceral larval migration, liver and lung lesions, and eosinophilic pneumonia in humans (23).

However trichinelliasis frequently occurs in extensive varieties of canids in Iran (24), in this study, no wild boars were infected.

In the three regions of the Mazandaran Province studied, only Sari city had no boars with helminthic infection. This may be due to the different climatic, geographical, and environmental conditions of this region, compared to the other regions. Sari city has comparatively lesser vegetation than the two other cities and is located in a flat area.

## Conclusion

Wild boars might play an important role in transmitting zoonotic parasites to humans. The abundance of these animals near human habitation creates favorable conditions for infection. Therefore, the risk of protozoan diseases increases in other animals and humans.
